# Efficacy of granulocyte colony stimulating factor in patients with severe alcoholic hepatitis with partial or null response to steroid (GRACIAH trial): study protocol for a randomized controlled trial

**DOI:** 10.1186/s13063-018-3092-7

**Published:** 2018-12-22

**Authors:** Yuri Cho, Youn Su Park, Hwi Young Kim, Won Kim, Heon Ju Lee, Dong Joon Kim

**Affiliations:** 1 Department of Internal Medicine, CHA Gangnam Medical Center, CHA University School of Medicine, Seoul, Republic of Korea; 2 Division of Gastroenterology and Hepatology, Department of Internal Medicine, Seoul Metropolitan Government Seoul National University Boramae Medical Center, 20, Boramae-ro 5-gil, Dongjak-gu, Seoul, 07061 Republic of Korea; 3 Department of Internal Medicine, College of Medicine, Ewha Womans University, Seoul, Republic of Korea; 4 Department of Internal Medicine, Yeungnam University College of Medicine, Yeungnam University Medical Center, 170, Hyeonchung-ro, Nam-gu, Daegu, 42415 Republic of Korea; 5 Department of Internal Medicine, Hallym University College of Medicine, Chuncheon Hallym University Medical Center, 153 Gyo-dong, Chuncheon-si, Gangwon-do 24253 Republic of Korea

**Keywords:** Alcoholic hepatitis, Prednisolone, G-CSF, Discriminant function

## Abstract

**Background:**

Alcoholic hepatitis (AH) has the most severe presentation among alcohol-related liver diseases. Corticosteroids are the most widely recommended treatment for severe AH. However, more innovative, refined treatment measures are required because of its high mortality despite corticosteroid treatment. This study aims to determine whether granulocyte colony stimulating factor (G-CSF) treatment increases short-term survival in patients with severe AH refractory to corticosteroid treatment.

**Methods/design:**

Patients with severe AH whose Maddrey’s discriminant function (MDF) score is ≥ 32 and who will be treated with prednisolone (40 mg/day) for 1 week will be screened. Among them, 190 subjects with a partial response (PR) (Lille score 0.16–0.56), and 78 subjects with a null response (NR) (Lille score ≥ 0.56) will be enrolled. Subjects with PR will be randomized to steroid plus placebo or steroid plus 12 G-CSF injections (5 μg/kg/day for 5 days followed by every 3 days) at a ratio of 1:1. Subjects with a NR will be randomized to the placebo or G-CSF group (1:1). Study subjects in the PR group will be treated with prednisolone for 28 days followed by dose tapering for an additional 2 weeks. The primary endpoint is the 2-month survival rate in the NR group and the 6-month survival rate in the PR group. Child–Turcotte–Pugh, model for end-stage liver disease score, and the change in the proportion of peripheral circulating CD34-positive cells will be analyzed as risk factors for mortality. Preliminary safety data for the initial 10 study subjects enrolled in the PR study will be assessed to determine whether the PR study would be continued, according to the G-CSF-mobilized, peripheral-blood stem cell donor assessment protocol of the National Marrow Donor Program.

**Discussion:**

We hypothesized that G-CSF would prolong short-term survival of patients with severe AH refractory to corticosteroid treatment. This is a proof-of-concept trial designed to assess the efficacy of Lille-score-guided G-CSF treatment. This trial is also designed to identify a special subgroup in whom G-CSF rescue treatment would improve liver function and prolong survival.

**Trial registration:**

ClinicalTrials.gov, NCT02442180. Prospectively registered on 13 May 2015.

**Electronic supplementary material:**

The online version of this article (10.1186/s13063-018-3092-7) contains supplementary material, which is available to authorized users.

## Background

Over several decades, South Korea has ranked as one of the developed countries with the largest consumption of alcohol in the world [1, 2]. Alcohol-related liver disease is a broad-spectrum disease entity, including hepatic steatosis, hepatitis, cirrhosis, and hepatocellular carcinoma, as one individual can have various progressive stages of liver damage. Among them, alcoholic hepatitis has the worst prognosis, with short-term mortality of 40% within 1 month of onset [3–5].

Severe alcoholic hepatitis (AH) is defined as Maddrey’s Discriminant Function (MDF) score ≥ 32 points or when the patient also had hepatic encephalopathy, which has a very poor prognosis and 28-day mortality of 30–50% if not treated [6, 7]. Severe AH has a higher short-term mortality rate than any other liver disease, including viral hepatitis and nonalcoholic fatty liver disease. Nonetheless, survival has not notably improved in severe AH, despite advances in medicine [8].

Corticosteroids (prednisolone 40 mg/day, 28 days) are the most widely recommended treatment for severe AH. Corticosteroid treatment is indicated for patients with severe AH who are expected to have a very poor prognosis, with a MDF score ≥ 32 points and model for end-stage liver disease (MELD) score > 21 points or hepatic encephalopathy [9, 10]. However, corticosteroids cannot be used when the patient also has upper gastrointestinal bleeding, renal failure, pancreatitis, or an uncontrolled infection [11, 12]. A recent meta-analysis of individual patient data from five randomized controlled trials demonstrated that the 28-day survival rate was significantly higher in the steroid group than in the placebo group (80.0% vs. 65.7%, *P* < 0.001). Thus, corticosteroids are a standard treatment in selected patients with AH and a MDF score ≥ 32 [7]. However, early recognition of non-response to corticosteroids (40% of the patients with severe AH who receive corticosteroid treatment) is essential to minimize unnecessary exposure to corticosteroids [13]. The short-term mortality of 20% despite corticosteroid treatment still desperately requires more advanced treatment measures [7].

To overcome the difficulties of exploring rescue therapies for steroid non-responders, several clinical trials have been conducted to increase the survival rate using granulocyte colony stimulating factor (G-CSF) in patients with severe AH [14, 15]. Although the standard treatment with corticosteroids may reduce necroinflammation in patients with AH, mortality remains strikingly high [16]. Therefore, if G-CSF treatment facilitates liver regeneration and enhances neutrophil function in patients with severe AH taking corticosteroids [17–19], it is expected to significantly improve the current standard treatment strategy.

The aim of this study is to test whether it is possible to increase the 2-month survival rate (null responder to steroids) or the 6-month survival rate (partial responder to steroids) using G-CSF treatment in patients with severe AH with high mortality despite corticosteroid treatment.

## Methods/design

### Study objectives

The aim of this study is to establish proof of concept whether patients with severe AH (MDF score ≥ 32) would benefit from G-CSF rescue therapy. The primary aim is to evaluate whether G-CSF treatment prolongs 6-month overall survival (OS) of patients with a partial response (PR) to steroids and 2-month OS of patients with a null response (NR) to steroids. Partial responders (0.16 < Lille score < 0.56) or null responders (Lille score ≥ 0.56) are identified based on the Lille score after 1 week of corticosteroid treatment (40 mg daily prednisolone dose). The secondary aims are to identify the risk factors in relation to mortality and the predictive factors associated with responses to standard corticosteroid treatment or rescue G-CSF therapy. Pre-allocation and post-allocation risk factors related to mortality include age, sex, the presence of ascites, infection, gastrointestinal bleeding, daily dose of alcohol consumed, the presence and period of abstinence, white blood cell count, hemoglobin, hematocrit, high sensitivity C-reactive protein, blood urea nitrogen, creatinine, aspartate aminotransferase (AST), alanine aminotransferase (ALT), gamma-glutamyl transferase, albumin, and prothrombin time. The secondary aims include improvements in liver function (Child–Turcotte–Pugh (CTP) score, MELD score, and chronic liver failure sequential organ failure assessment (CLIF-SOFA)( score), changes in the percentage of CD34-positive cells in peripheral blood, and changes in hepatic histological findings.

### Sample size

The 1-month OS rate in patients with severe AH undergoing steroid therapy has been reported as 80% and that in a control group as 66% [7]. Moreover, the 6-month OS rate of steroid responders has been reported as 85% and that of non-responders as 25% [13]. A previous, large-scale, randomized study compared co-administration of steroid and pentoxifylline with steroid monotherapy. The 6-month survival rate of the complete responders in the steroid monotherapy group was 90.5%, whereas the 6-month survival rate of partial responders was 82% [20]. The 2-month survival rate was 66% in the G-CSF group and 26% in the control group in a previous study conducted in patients with hepatic failure, in whom AH was responsible for approximately 60% of cases of hepatic failure [14]. Additionally, the 2-month survival rate of null responders was 42% [21]. Based on these data, the sample size was estimated using PASS 11.0 software (NCSS, Kaysville, UT, USA).

We hypothesized that there would be a significant difference in 6-month OS between steroid therapy and steroid plus G-CSF therapy in patients with a PR to steroids (82% vs. 90.5%). A two-sided sample size calculation with power of 0.8, significance level of 0.05, and estimated dropout rate of 10% revealed that at least 190 patients should be included in the PR study: 95 patients in the steroid plus placebo group and 95 in the steroid plus G-CSF group.

We hypothesized that there would be a significant difference in 2-month OS between placebo therapy and G-CSF therapy (42% vs. 66%) for patients with a NR to steroids. A two-sided sample size calculation with power of 0.8, significance level of 0.05, and estimated dropout rate of 10% revealed that at least 78 patients should be included in the NR study: 39 patients in the placebo group and 39 in the G-CSF group.

### Trial design

**Fig. 1:**
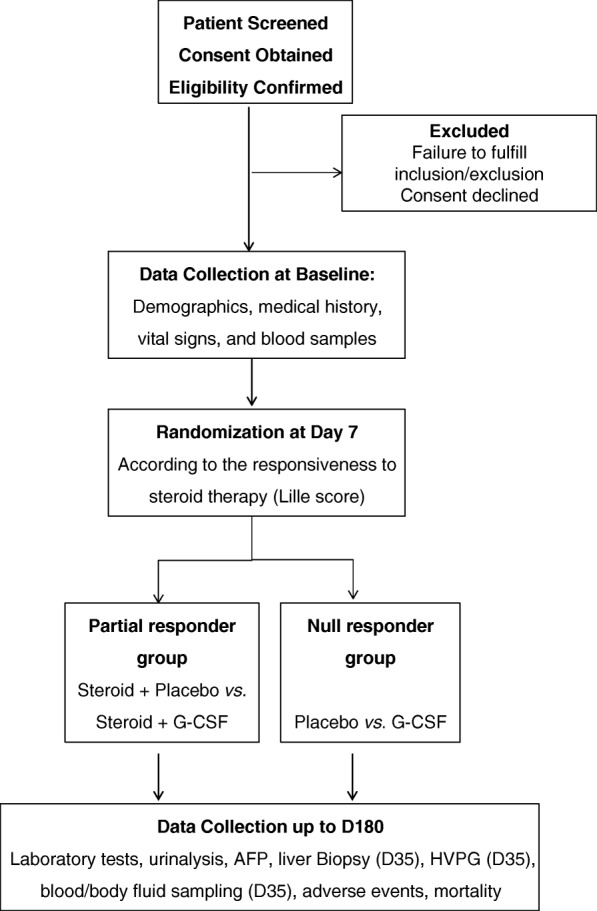
Flowchart for study participants. Abbreviations: G-CSF, granulocyte colony stimulating factor; AFP, alpha-fetoprotein; HVPG, hepatic venous pressure gradient; D, day

The current study is a prospective, double-blind, multicenter (15 centers), randomized, placebo-controlled trial with two parallel subgroups (PR and NR groups) (Fig. 1). We will evaluate the PR (Lille score 0.16–0.56) and NR (Lille score ≥ 0.56) by calculating the Lille score after 1 week of steroid therapy in patients who meet the eligibility criteria. Partial responders are expected to have the OS benefits of steroid therapy, thereby, the subjects will be randomly assigned to either the steroid-placebo co-administration group or the steroid-G-CSF co-administration group (1:1). Study subjects in the PR group will be treated with prednisolone 40 mg/day for 28 days followed by dose tapering.

Steroid therapy will be discontinued in the NR group because the clinical benefits from steroid therapy are not proven and there was concern over adverse effects of steroid therapy in the NR group. When liver transplantation is not feasible for NRs, they will be randomly assigned to either the placebo group or the G-CSF group (1:1).

A preliminary study including five subjects each from the steroid–G-CSF versus steroid–placebo groups in the PR group will be conducted to assess safety data according to the adverse event assessment criteria of the National Marrow Donor Program (NMDP) filgrastim-mobilized, peripheral-blood stem cell donor assessment for the solid validation on the safety of steroid-G-CSF co-administration [22]. The independent Data Safety Monitoring Board (DSMB) committee (a hepatologist, an oncologist, a gastroenterologist, and a biostatistician, who do not participate in this trial) will analyze the preliminary safety data according to the NMDP safety assessment criteria. The data from the preliminary study will be unblinded only to the DSMB committee, therefore will be included in the main study results. The trial follows the recommendations for interventional trials guidelines (SPIRIT; see Additional file 1).

### Interventions

**Fig. 2:**
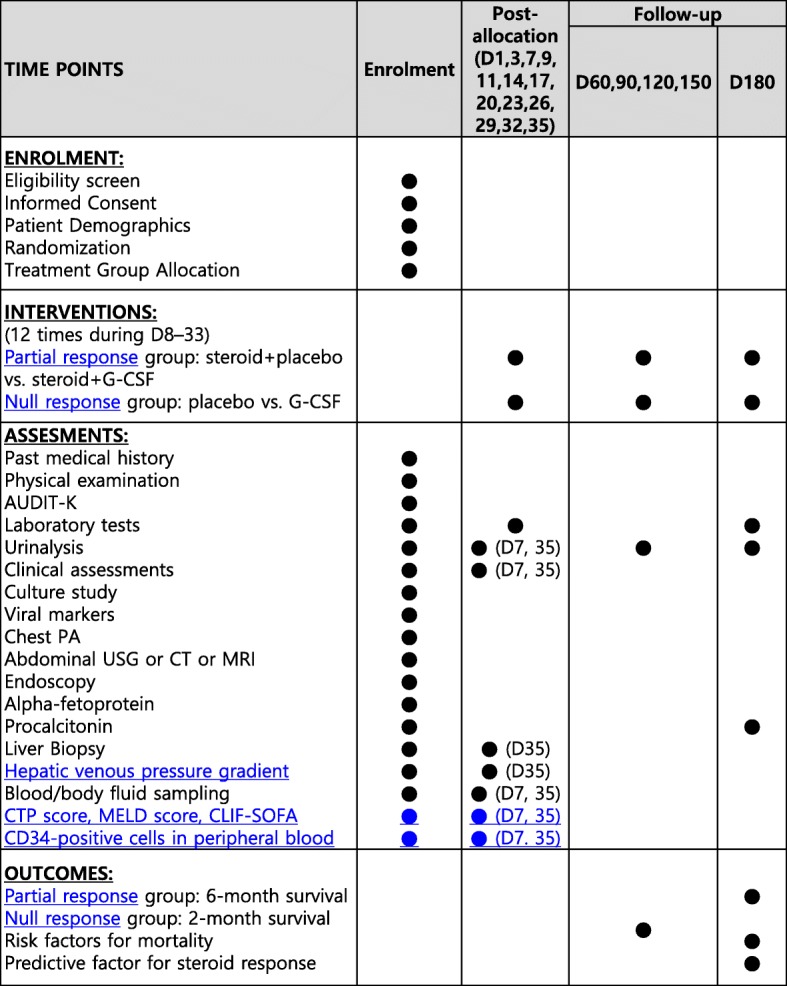
Study schedule of enrollment, intervention, and assessment. Abbreviations: G-CSF, granulocyte colony stimulating factor; AUDIT-K, Alchol Use Disorder Identification Test-Korea; USG, ultrasonography; CT, computed tomography; MRI, magnetic resonance imaging; CTP, Child–Turcotte–Pugh, MELD, model for end-stage liver disease; CLIF-SOFA, chronic liver failure sequential organ failure assessment; D, day

All study subjects are started on corticosteroid as standard therapy if they have no contraindications to corticosteroid treatment (prednisolone 40 mg daily); partial responders will have their dose reduced after 28 days of administration, whereas null responders will stop the drug after 7 days administration. When oral dosing is not tolerable, 32 mg methylprednisolone will be administered via intravenous injection daily, which is a dosage with equivalent efficacy. G-CSF will be subcutaneously injected at a dosage of 5 μg/kg body weight daily for 5 days from the next day of randomization in the PR and NR groups. Thereafter, it will be administered at the same dosage every 3 days, for a total of 12 times (days of G-CSF administration, day 8–12, 15, 18, 21, 24, 27, 30, and 33) (Fig. 2).

### Study drug

G-CSF (Leucostim® Injection, Dong-A ST Co., Ltd., Seoul, Korea, nomenclature of component filgrastim) is regarded as an investigational medicinal product for this study. The dose and volume of G-CSF will be determined on the first day according to the body weight of each subject (Table 1). G-CSF will be subcutaneously administered 12 times (daily for 5 days, followed by every 3 days for 7 times). Normal saline (JW Pharmaceutical, Seoul, Korea) will be used as a placebo at a maximal volume of 1.2 mL.

**Table 1:** Dose of G-CSF according to the body weight of each subject

Bodyweight (kg)	Dose of G-CSF (μg)	Volume (mL)
40–44	200	0.8
45–49	225	0.9
50–54	250	1.0
55–59	275	1.1
≥60	300	1.2

*G-CSF* granulocyte colony stimulating factor

### Inclusion criteria

The inclusion criteria are as follows:

- Age > 20 years and < 80 years
- History of clinically significant amount of alcohol intake (≥ 50 g daily for men, and ≥ 40 g daily for women over the last 2 months)
- MDF during the screening period = [4.6 × (patient’s prothrombin time, sec − control prothrombin time, sec)] + serum bilirubin level (mg/dL) ≥ 32
- New occurrence of jaundice within the last 3 months when a liver biopsy could not be performed, (total serum bilirubin level > 5 mg/dL), or pathological transjugular liver tissue biopsy results consistent with alcoholic hepatitis (hepatocellular ballooning and polymorphonuclear leukocytic infiltration)
- After satisfying criteria 1–4, Lille score > 0.16 on day 7 after daily administration of 40 mg prednisolone (or intravenous methylprednisolone at the equivalent efficacy dose in the case of oral intolerability)

### Exclusion criteria

The exclusion criteria are as follows:

- Positive for the hepatitis B surface antigen, anti-hepatitis C antibody, or anti-human immunodeficiency virus antibody
- Malignant neoplasm including hepatocellular carcinoma
- AST > 500 IU/L or ALT > 300 IU/L
- Portal vein thrombosis, hemochromatosis, autoimmune hepatitis, Wilson’s disease, or alpha-1-antitrypsin deficiency
- Pregnant or breastfeeding women or those who refuse to use or cannot use contraceptives
- History of hypersensitivity to G-CSF injection
- Hypovolemic shock due to gastrointestinal bleeding at the time of hospitalization, or in need of a transfusion of more than three units of packed red blood cells, or MDF elevated from < 32 points to ≥ 32 points due to gastrointestinal bleeding
- Sepsis or uncontrolled acute infection
- Hepatic encephalopathy grade 3–4
- Previous history treatment with corticosteroids or pentoxifylline within the past 3 months
- Myeloblasts observed in peripheral blood
- Severe comorbidities (type 1 hepatorenal syndrome or serum creatinine > 2.5 mg/dL at the time of screening, heart failure, lung disease, mental illness, or acute pancreatitis)
- Refusal to participate in the clinical trial

### Consent

The investigator will explain the details of this clinical trial to potential study subjects, and provide sufficient time for them to consider whether they will participate in the study. Voluntary informed consent will be obtained from the study subjects in a written form. Consent will be documented as being signed and dated by the study subject on the informed consent form. Consent will be obtained in a written form from a parent or a legal guardian or a legally acceptable representative when a study subject is legally incompetent.

### Randomization and blinding procedures

The study participants will be given a unique identification number, and they will be assigned to either the study-drug group or the control-placebo group. Randomization will be conducted using a web-based randomization program constructed by means of the blocked randomization method. The web-based randomization will be managed by the Medical Research Collaborating Center of Seoul National University Hospital (https://mrcc.snuh.org/) and stratification variables are individual centers and whether or not liver biopsy was performed.

The placebo and the study drug will be managed and supplied by the clinical pharmacy at each institution. A subject identification number will be given to each study subject in accordance with the randomization table, and the clinical pharmacy will provide the placebo or the study drug with the study subjects and the investigators blinded to allocation.

### Safety

The principal investigator will observe adverse events (AEs) and all AEs spontaneously reported by the study subjects. The principal investigator will assess all AEs for seriousness, causality, severity, and for expectedness if the AE is related to the study drug. All AEs will be assessed by the principal investigator as possibly, probably, or definitely related to the study drug and all serious adverse events (SAEs) that occur during the study period will be followed until they are resolved or clearly determined to be due to a patient’s stable or chronic conditions or intercurrent illness. Reporting will follow the filgrastim-mobilized, peripheral blood stem cell donor assessment of NMDP [23].

A SAE is defined as an AE that:

- Results in death
- Results in life-threatening events
- Results in persistent or significant disability or incapacity involving a congenital anomaly or birth defect
- Represents any other important medical event that carries a real but not a hypothetical risk of one of the outcomes listed above

### Data collection and management

An electronic clinical record form (eCRF; see Additional file 2) will be used to collect the data on each study subject. The study subjects will be identified on the eCRF through their unique trial identifier, allocated at the time of recruitment. Data will be coded at various stages during the study period. Data security and storage will follow the data management plan of the Data Safety Monitoring Board (DSMB). The DSMB plan will be developed by referring to the guidelines of the World Health Organization [24].

### Statistical analyses

Intention-to-treat analysis will be used as the main assessment of efficacy. Per-protocol will be analyzed for the primary efficacy analysis (i.e., OS), as well as to ensure the stability of analytical outcomes. The OS is defined as the period from enrollment to death or the last follow-up date, and will be analyzed using Kaplan–Meier estimates. To test the difference in OS between the G-CSF and control groups, a log-rank test will be used, and the significance level will be 5% (two-sided test).

We will summarize the demographic and other baseline characteristics of the study participants. Continuous variables will be presented as mean ± standard deviations or median (interquartile range). The two-sample *t* test or Wilcoxon’s rank sum test will be used to compare the groups according to the normal distribution. Categorical variables will be presented as frequency and the percentage. Pearson’s chi-square test or Fisher’s exact test will be used to compare the groups. Changes in the percentage of CD34-positive cells in peripheral blood and liver tissues at 1 month of treatment will be compared between the groups using the two-sample *t*-test or Wilcoxon’s rank sum test. In the PR group, the changes in CTP score, MELD score, and CLIF-SOFA score at 1 week and 1, 2, 3, 4, 5, and 6 months will be compared between the groups (steroid + placebo versus steroid + G-CSF) using the two-sample *t* test or Wilcoxon’s rank sum test. In the NR group, the changes in CTP score, MELD score, and CLIF-SOFA score at 1 week, 1 month, and 2 months will be compared between the groups (placebo vs. G-CSF) using two-sample *t* test or Wilcoxon’s rank sum test. In addition, repeated measures analysis of variance (ANOVA) will be performed to analyze the difference in repeated measures between the groups. OS of the subgroups according to the alcoholic hepatitis histologic score (AHHS) will be calculated by the Kaplan–Meier method and will be compared by log-rank test. Multivariate Cox regression analysis will be used to evaluate the independent variables (*P* < 0.05) in the whole study sample, the PR group, and the NR group. To determine the mortality prediction model, the cutoff value using area under the receiver operating characteristic curve (AUROC) will be calculated, and sensitivity and specificity will be calculated. The discrimination function of the model will be evaluated by the Kaplan–Meier method according to the estimated cut-off value. All analyses will be performed using SPSS Statistics software version 20.0 (IBM Corp., Armonk, NY, USA) and SAS version 9.2 software (SAS Institute, Cary, NC, USA).

## Discussion

The purpose of this trial is to establish proof of concept of the efficacy of G-CSF in patients with histologically confirmed or clinically suspected, severe AH (MDF score ≥ 32). Patients will be screened and enrolled into the PR (Lille score 0.16–0.56) and NR (Lille score ≥ 0.56) groups by calculating the Lille score after 1 week of steroid therapy. Steroid therapy will be discontinued in null responders upon allocation to the NR group. They will be randomly assigned to either the placebo group or the G-CSF group (1:1). Partial responders are expected to have a survival benefit from steroid therapy, thereby the subjects will be randomly assigned to either the steroid-placebo co-administration group or the steroid-G-CSF co-administration group (1:1). Corticosteroids might reduce necroinflammation in the liver, but a high mortality rate is still noted in patients with severe who are AH receiving steroid therapy. Combining G-CSF with corticosteroid treatment might promote liver regeneration and improve neutrophil function, leading to the amelioration of clinical outcomes.

The Korean Association for the Study of the Liver clinical practice guidelines for the management of alcoholic liver disease (2013) recommend liver transplantation when there is no remarkable reduction in jaundice (Lille model score ≥ 0.56) 1 week after steroid treatment is initiated [25]. However, in addition to a shortage of donor organs, it calls for pre-transplant 6-month abstinence from alcohol as a prerequisite for liver transplantation in most transplant centers; therefore, it is practically difficult to follow the recommendation of “considering a liver transplant” as viable.

The 6-month survival rate is higher in patients with severe AH who are receiving pentoxifylline than in those treated with placebo [26]. Pentoxifylline can be considered an alternative therapy for patients with severe AH and contraindication to corticosteroid treatment [27, 28]. A previous study reported the therapeutic efficiency of pentoxifylline in non-responders switching from corticosteroids [16]. Among the non-responders to corticosteroids, the investigators compared the 2-month survival rate in patients in whom corticosteroids were replaced with pentoxifylline at the early stage with that in those who had continuously taken corticosteroids; however, the 2-month survival rates were 35.5% and 31.0%, respectively (*P* value not significant) [16]. Therefore, the switch to pentoxifylline is not clinically beneficial in non-responders to corticosteroids.

It is essential to develop a new treatment modality to overcome therapeutic refractoriness or resistance and improve survival of patients with severe AH who are refractory to steroid therapy. Some studies have reported that G-CSF stimulates proliferation of hepatocytes in patients with alcoholic AH, cirrhosis, and liver failure, by mobilizing CD34-positive stem cells to the liver [14, 15]. These studies enrolled a small number of subjects and the follow-up period was only 1 month. However, they suggest G-CSF may be beneficial in improving long-term survival in patients with severe AH. Therefore, we hypothesize that G-CSF treatment might prolong the survival of patients with severe AH who are refractory to corticosteroid treatment. This is the first proof-of-concept trial designed to assess the efficacy of G-CSF according to the response to corticosteroids. This large-scale, multicenter, randomized controlled trial is also designed to find a special subgroup of patients in whom G-CSF effectively works to improve liver function and prolong survival.

## Trial status

This trial is ongoing and actively recruiting. Recruitment started on 13 May 2015. Completion is anticipated on 31 December 2020.

## Additional files

Additional file 1: SPIRIT checklist. (DOC 123 kb)

Additional file 2: Case Report Form. (PDF 911 kb)

## Abbreviations

AFP: Alpha-fetoprotein
AH: Alcoholic hepatitis
AHHS: Acoholic hepatitis histologic sore
ALT: Alanine aminotransferase
ANOVA: Analysis of variance
AST: Aspartate aminotransferase
AUROC: Area under the receiver operating characteristic curve
CBC: Complete blood cell count
CLIF-SOFA: Chronic liver failure sequential organ failure assessment
CR: Complete response
DF: Discriminant function
EASL: European Association for the Study of the Liver
eCRF: Electronic clinical record form
G-CSF: Granulocyte colony stimulating factor
HVPG: Hepatic venous pressure gradient
ITT: Intention to treat
MDF: Maddrey’s discriminant function
MELD: Model for end-stage liver disease
NMDP: National Marrow Donor Program
NR: Null response
OS: Overall survival
PR: Partial response

## References

[1] Rehm J, Samokhvalov AV, Shield KD (2013). Global burden of alcoholic liver diseases. J Hepatol.

[2] Jang JY, Kim DJ (2018). Epidemiology of alcoholic liver disease in Korea. Clin Mol Hepatol.

[3] Lucey MR, Mathurin P, Morgan TR (2009). Alcoholic hepatitis. N Engl J Med.

[4] Mathurin P, Duchatelle V, Ramond MJ (1996). Survival and prognostic factors in patients with severe alcoholic hepatitis treated with prednisolone. Gastroenterology.

[5] Kim WR, Brown RS, Terrault NA, El-Serag H (2002). Burden of liver disease in the United States: summary of a workshop. Hepatology.

[6] Dominguez M, Rincon D, Abraldes JG (2008). A new scoring system for prognostic stratification of patients with alcoholic hepatitis. Am J Gastroenterol.

[7] Mathurin P, O’Grady J, Carithers RL (2011). Corticosteroids improve short-term survival in patients with severe alcoholic hepatitis: meta-analysis of individual patient data. Gut.

[8] Liangpunsakul S (2011). Clinical characteristics and mortality of hospitalized alcoholic hepatitis patients in the United States. J Clin Gastroenterol.

[9] Maddrey WC, Boitnott JK, Bedine MS, Weber FL, Mezey E, White RI (1978). Corticosteroid therapy of alcoholic hepatitis. Gastroenterology.

[10] Dunn W, Jamil LH, Brown LS (2005). MELD accurately predicts mortality in patients with alcoholic hepatitis. Hepatology.

[11] European Association for the Study of L (2012). EASL clinical practical guidelines: management of alcoholic liver disease. J Hepatol.

[12] O’Shea RS, Dasarathy S, McCullough AJ, Practice Guideline Committee of the American Association for the Study of Liver Disease and Practice Parameters Committee of the American College of Gastroenterology (2010). Alcoholic liver disease. Hepatology.

[13] Louvet A, Naveau S, Abdelnour M (2007). The Lille model: a new tool for therapeutic strategy in patients with severe alcoholic hepatitis treated with steroids. Hepatology.

[14] Garg V, Garg H, Khan A (2012). Granulocyte colony-stimulating factor mobilizes CD34(+) cells and improves survival of patients with acute-on-chronic liver failure. Gastroenterology.

[15] Spahr L, Lambert JF, Rubbia-Brandt L (2008). Granulocyte-colony stimulating factor induces proliferation of hepatic progenitors in alcoholic steatohepatitis: a randomized trial. Hepatology.

[16] Louvet A, Diaz E, Dharancy S (2008). Early switch to pentoxifylline in patients with severe alcoholic hepatitis is inefficient in non-responders to corticosteroids. J Hepatol.

[17] Gaia S, Smedile A, Omede P (2006). Feasibility and safety of G-CSF administration to induce bone marrow-derived cells mobilization in patients with end stage liver disease. J Hepatol.

[18] Singh V, Sharma AK, Narasimhan RL, Bhalla A, Sharma N, Sharma R (2014). Granulocyte colony-stimulating factor in severe alcoholic hepatitis: a randomized pilot study. Am J Gastroenterol.

[19] Piscaglia AC, Shupe TD, Oh SH, Gasbarrini A, Petersen BE (2007). Granulocyte-colony stimulating factor promotes liver repair and induces oval cell migration and proliferation in rats. Gastroenterology.

[20] Mathurin P, Louvet A, Duhamel A (2013). Prednisolone with vs without pentoxifylline and survival of patients with severe alcoholic hepatitis: a randomized clinical trial. JAMA.

[21] Mathurin P, Moreno C, Samuel D (2011). Early liver transplantation for severe alcoholic hepatitis. N Engl J Med.

[22] National Marrow Donor Program (2014). Filgrastim mobilized PBSC days one, two, three and four donor assessment.

[23] Pulsipher MA, Chitphakdithai P, Miller JP (2009). Adverse events among 2408 unrelated donors of peripheral blood stem cells: results of a prospective trial from the National Marrow Donor Program. Blood.

[24] World Health Organization (2004). Operational guidelines for the establishment and functioning of data & safety monitoring boards.

[25] Korean Association for the Study of the Liver (2013). KASL clinical practice guidelines: management of alcoholic liver disease. Clin Mol Hepatol.

[26] Akriviadis E, Botla R, Briggs W, Han S, Reynolds T, Shakil O (2000). Pentoxifylline improves short-term survival in severe acute alcoholic hepatitis: a double-blind, placebo-controlled trial. Gastroenterology.

[27] Parker R, Armstrong MJ, Corbett C, Rowe IA, Houlihan DD (2013). Systematic review: pentoxifylline for the treatment of severe alcoholic hepatitis. Aliment Pharmacol Ther.

[28] Whitfield K, Rambaldi A, Wetterslev J, Gluud C. Pentoxifylline for alcoholic hepatitis. Cochrane Database Syst Rev. 2009;(4):CD007339. 10.1002/14651858.CD007339.pub2 PMC6769169 19821406

