# Supplementary material for: Efficacy of granulocyte colony stimulating factor in patients with severe alcoholic hepatitis with partial or null response to steroid (GRACIAH trial): study protocol for a randomized controlled trial
Source: Trials. 2018 Dec 22;19:696. doi: 10.1186/s13063-018-3092-7 (PMC6303849; doi:10.1186/s13063-018-3092-7)
Supplement: Supplementary file 2 — Case Report Form. (PDF 911 kb) [file 13063_2018_3092_MOESM2_ESM.pdf]

Screening Study Participants No. _ _ S _ _ _ Initial of Name:

Date of birth: YYYY-MM-DD

Date of admission (the 1st date to ER or ward) : YYYY-MM-DD

Age: years

Sex: □ Male □ Female

Bwt: Kg

Ht: cm

BMI:

Screening (MDF≥32): YYYY-MM-DD

AUDIT-K ☐ Yes ☐ No

History Taking

Major	Minor	Result
Alcohol	Type of drinking	☐ Soju<br>☐ Bear<br>☐ Makgeolli<br>☐ Whisky<br>☐ Wine
	Number of drinking per week	Times/week
	Dose of Drinking for one time	
Smoking		PPD

Medical History

☐ None

Disease/Surgery	Start Year (YYYY)	On-going	On Treatment
		☐Yes ☐No	☐Yes ☐No
		☐Yes ☐No	☐Yes ☐No
		☐Yes ☐No	☐Yes ☐No
		☐Yes ☐No	☐Yes ☐No
		☐Yes ☐No	☐Yes ☐No
		☐Yes ☐No	☐Yes ☐No

		☐Yes ☐No	☐Yes ☐No
		☐Yes ☐No	☐Yes ☐No
		☐Yes ☐No	☐Yes ☐No

Concomitant medication (which is used after the consent to clinical trial)

: Only the drugs of clinical significance are recorded by the clinician. (Ex. Fluid therapy is excluded)

☐ None

Medication	Dose	Start Date	On-going	End Date

☐Dopamine (dose__ μg/min), Start Date YYYY-MM-DD, End Date YYYY-MM-DD

☐Dobutamine (dose__ μg/min _), Start Date YYYY-MM-DD, End Date YYYY-MM-DD

☐Terlipressin (dose__mg_), Start Date YYYY-MM-DD, End Date YYYY-MM-DD

☐Epinephrine (dose__ μg/min), Start Date YYYY-MM-DD, End Date YYYY-MM-DD

☐Norepinephrine (dose__μg/min_), Start Date YYYY-MM-DD, End Date YYYY-MM-DD

☐Antibiotics ☐Yes (Drug Name:), Start Date YYYY-MM-DD, End Date YYYY-MM-DD

☐No

Date of test (YYYY-MM-DD):

Complete blood count (CBC)

Item	Result	If abnormal, CS NCS judgment
WBC($\chi 10^3/\mu L$)		☐ CS ☐ NCS
Neutrophil (%)		☐ CS ☐ NCS
Lymphocyte (%)		☐ CS ☐ NCS
Monocyte (%)		☐ CS ☐ NCS
Hemoglobin(g/dL)		☐ CS ☐ NCS
Platelet($\chi 10^3/\mu L$)		☐ CS ☐ NCS

Routine Chemistry

Item	Result	If abnormal, CS NCS judgment
Inorganic P(mg/dl)		☐ CS ☐ NCS
Glucose(mg/dl)		☐ CS ☐ NCS
BUN(mg/dl)		☐ CS ☐ NCS
Creatinine(mg/dl)		☐ CS ☐ NCS
GFR (mL/min)		☐ CS ☐ NCS
Uric acid(mg/dl)		☐ CS ☐ NCS
Cholesterol(mg/dl)		☐ CS ☐ NCS
Calcium(mg/dl)		☐ CS ☐ NCS
Total Protein(g/dl)		☐ CS ☐ NCS
Albumin(g/dl)		☐ CS ☐ NCS
Total bilirubin(mg/dl)		☐ CS ☐ NCS

Alk.Phosphatase(IU/L)		☐ CS ☐ NCS
AST(SGOT)(IU/L)		☐ CS ☐ NCS
ALT(SGPT)(IU/L)		☐ CS ☐ NCS
GGT (IU/L)		☐ CS ☐ NCS
Na (mmol/L)		☐ CS ☐ NCS
K(mmol/L)		☐ CS ☐ NCS
CRP(mg/dl)		☐ CS ☐ NCS

Coagulation test

Item	Result	If abnormal, CS NCS judgment
PT(Sec)	Sec	☐ CS ☐ NCS
PT (%)	%	☐ CS ☐ NCS
PT (INR)		☐ CS ☐ NCS

Urinalysis, ascites, culture study, stool lab

Specimen	Item	Result	If abnormal, CS NCS judgment
Urinalysis	WBC(/HPF)		☐ CS ☐ NCS
	RBC(/HPF)		☐ CS ☐ NCS
	cast		☐ CS ☐ NCS
	bacteria		☐ CS ☐ NCS
	bilirubin		☐ CS ☐ NCS
	nitrite		☐ CS ☐ NCS
	Na/K/Cl(mmol/L)		☐ CS ☐ NCS
Ascites ☐Not	WBC(absolute count)		☐ CS ☐ NCS
	Neutrophil(absolute count)		☐ CS ☐ NCS

done<br>☐Done	Lymphocyte(absolute count)		☐ CS ☐ NCS
	Monocyte(absolute count)		☐ CS ☐ NCS
	RBC(absolute count)		☐ CS ☐ NCS
	Protein(mg/dl)		☐ CS ☐ NCS
	Glucose(mg/dl)		☐ CS ☐ NCS
	Amylase(U/L)		☐ CS ☐ NCS
	Albumin(g/dl)		☐ CS ☐ NCS
Culture study	Blood	☐Not done<br>☐Positive (Name of bacteria:)<br>☐Negative	☐ CS ☐ NCS
	Ascites	☐Not done<br>☐Positive (Name of bacteria:)<br>☐Negative	☐ CS ☐ NCS
	Urine	☐Not done<br>☐ Positive (Name of bacteria:)<br>☐Negative	☐ CS ☐ NCS
	Stool	☐Not done<br>☐ Positive (Name of bacteria:)<br>☐Negative	☐ CS ☐ NCS
Stool	parasite	☐Not done	☐ CS ☐ NCS

		( )	
	ova	☐Not done<br>( )	☐ CS ☐ NCS
	occult blood	☐Not done<br>( )	☐ CS ☐ NCS

Samples for research

☐ Blood ☐ Urine ☐ Ascites ☐ Stool ☐ Liver tissue ☐ No

Clinical evaluation

Item	Result	If abnormal, CS NCS judgment
Systolic BP (mmHg)		
Diastolic BP (mmHg)		
Pulse rate (bpm)		
PaO_2<br>☐Y ☐N	( )mmHg	
SpO_2<br>☐Y ☐N	( )%	
O_2 supply	☐No ☐Yes ( ) L/min<br>☐ facial mask ☐ nasal prong	
Mechanical ventilation	☐Yes (FiO_2:) ☐No	
Respiratory rate (/min)		
Body temperature (°C)		
Infection at admission	☐Spontaneous bacteremia	

	□SBP<br>□Lower respiratory tract infection<br>□C. difficile<br>□bacterial enterocolitis<br>□skin infection<br>□UTI<br>□intraabdominal infection<br>□secondary bacterial peritonitis	
Gastrointestinal hemorrhage<br>□Not done □Done	□No □Yes(□Esophageal varix<br>□Gastric varix<br>□Portal hypertensive gastropathy<br>□Gastric ulcer<br>□Duodenal ulcer<br>□Mallory-Weiss<br>□Angiodysplasia<br>□etc(______)	
Encephalopathy	□No □Yes (grade__ [1-4])	1) Grade 0: Subclinical<br>2) Grade 1: depression, mania, sleep disturbance<br>3) Grade 2: somnolence, personality change, flapping

		tremor<br>4) Grade 3: Most of the time sleep but you can wake up<br>5) Grade 4: coma
Dialysis / renal replacement therapy	☐Not done ☐Done	

Other blood test : ☐ Yes ☐ No

Date of test: (YYYY-MM-DD) :

Image, tissue, other blood test

Item	Result	If abnormal, check CS NCS
HBsAg	☐negative ☐positive	☐ CS ☐ NCS
Anti-HCV	☐negative ☐positive	☐ CS ☐ NCS
Anti-HIV	☐negative ☐positive	☐ CS ☐ NCS
Chest PA<br>☐Y ☐N<br>(YYYY-MM-DD)	☐Normal ☐Abnormal (impression:_____)	☐ CS ☐ NCS
Abdomen USG or CT or MRI ☐Y ☐N<br>(YYYY-MM-DD)	☐Normal<br>☐Abnormal (impression:______)<br>Ascites ☐Yes ☐No	
Endoscopy<br>☐Y ☐N<br>(YYYY-MM-DD)	☐Normal ☐Abnormal<br>☐Esophageal varix: F1/2/3, RCS(+/-)<br>☐Gastric varix: F1/2/3, RCS(+/-)<br>☐Portal hypertensive gastropathy<br>☐Gastric ulcer	

	☐Duodenal ulcer<br>☐Mallory-Weiss<br>☐Angiodysplasia<br>☐etc______	
AFP<br>☐Y ☐N<br>(YYYY-MM-DD)	☐ IU/ml<br>☐ ng/ml	☐ CS ☐ NCS
Procalcitonin (ng/ml)<br>☐Y ☐N<br>(YYYY-MM-DD)		
HVPG<br>☐Y ☐N<br>(YYYY-MM-DD)	( )mmHg	
Histologic scoring (transjugular or percutaneous liver biopsy)<br>☐Y ☐N<br>(YYYY-MM-DD)	hepatocellular damage/ballooning (0=mild; 1=marked)	
	Mallory bodies (0=absent; 1=present)	
	Polymorphonuclear (PMN) infiltration (0=mild; 1=moderate/severe)	
	Steatosis (0, <33%; 1, 33 - 66%; 2, >66%)	
	Lobular fibrosis (0=no fibrosis or zone 3; 1=fibrosis in zone 2+3; 2=panlobular fibrosis),	
	Fibrosis stage (0=no fibrosis or portal fibrosis; 1= expansive periportal fibrosis; 2= bridging fibrosis and	

	cirrhosis)	
	Megamitochondria (0=no; 1=yes)	
	Bilirubinostasis (0=none; 1=hepatocellular bilirubinostasis; 2=canalicular or ductular bilirubinostasis; 3=hepatocellular + canalicular or ductular bilirubinostasis)	

Eligibility Evaluation

**Criteria of Inclusion**	**Yes**	**No**
1. Age > 20 years, < 80 years	❑	❑
2. History of clinically significant amount of alcohol intake (≥50 g daily for men, and ≥40 g daily for women over the last 2 months)	❑	❑
3. MDF during the screening period = [4.6 × (patient's prothrombin time, sec − control prothrombin time, sec)] + serum bilirubin level (mg/dL) ≥32	❑	❑
4. Newly occurred jaundice within the last 3 months when a liver biopsy could not be performed, (total serum bilirubin level >5 mg/dL), or pathological findings of transjugular liver tissue biopsy consistent with alcoholic hepatitis (hepatocellular ballooning and polymorphonuclear leukocytic infiltration)	❑	❑

5. After satisfying criteria nos. 1–4, Lille score >0.16 on day 7 after daily administration of 40 mg prednisolone (or intravenous methylprednisolone of the equivalent efficacy dose in case of oral intolerability)	❑	❑
▶ If any of the above criteria falls into 'No', you will not be able to participate in random assignments.		

**Criteria of Exclusion**	**Yes**	**No**
1. Positive for the hepatitis B surface antigen, anti-hepatitis C antibody, or anti-human immunodeficiency virus antibody	❑	❑
2. Malignant neoplasm including hepatocellular carcinoma	❑	❑
3. Portal vein thrombosis, hemochromatosis, autoimmune hepatitis, Wilson's disease, or alpha-1-antitrypsin deficiency	❑	❑
4. Pregnant or breastfeeding women or those who refuse to use or cannot use contraceptives	❑	❑
5. History of hypersensitivity to G-CSF injection	❑	❑
6. Hypovolemic shock due to gastrointestinal bleeding at the time of hospitalization, or in need of a transfusion of more than 3 units of packed red blood cells, or MDF is elevated from <32 points to ≥32 points due to gastrointestinal bleeding	❑	❑
7. Sepsis or uncontrolled acute infection	❑	❑
8. Hepatic encephalopathy grade 3–4	❑	❑
9. Patients with a previous treatment history of corticosteroids or pentoxifylline within the past 3 months	❑	❑
10. Myeloblasts observed in peripheral blood	❑	❑
11. Severe comorbidities (type 1 hepatorenal syndrome or serum creatinine	❑	❑

>2.5 mg/dL at the time of screening, heart failure, lung disease, mental illness, and acute pancreatitis)		
12. Refusal to participate in the clinical trial	❑	❑
▶ If any of the above criteria falls into 'Yes', you will not be able to participate in random assignments.		
**Assessment of suitability of subjects**		
Is this study participant trial eligible for this clinical? ❑ Yes ❑ No →Reason: ________		
**Signature / Date**		

**Writer**		**Checker**	

Screening failure

**Date of screening failure (before randomization)**	(YYYY-MM-DD)
**Reason** → check one point	❑ Ineligibility for inclusion/exclusion criteria<br>❑ Liver transplantation<br>❑ Adverse event<br>❑ Non-fulfilment for the trial plan

	❑ Request for the study participants<br>❑ Follow-up loss<br>❑ etc → reason:_______________________________________		
**Signature / Date**			/
**Writer**		**Checker**	

Drug

Steroid<br>☐ Prednisolone<br>☐ methylprednisolone	Start date (YYYY-MM-DD) ,<br>Dose (mg)<br>End date (YYYY-MM-DD)
Pentoxifylline<br>☐Yes ☐No	Start date (YYYY-MM-DD) ,<br>Dose (mg)<br>End date (YYYY-MM-DD)
N-acetylcysteine<br>☐Yes ☐No	Start date (YYYY-MM-DD) ,<br>Dose (mg)<br>End date (YYYY-MM-DD)

Partial responder or Null responder

G-CSF or placebo injection ( ) ug/kg (Date of Injection: day 8-12, 15, 18, 21, 24, 27, 30, and 33).

1st Dose : (YYYY-MM-DD)
2nd Dose : (YYYY-MM-DD)
3rd Dose : (YYYY-MM-DD)
4th Dose :(YYYY-MM-DD)
5th Dose : (YYYY-MM-DD)
6th Dose : (YYYY-MM-DD)
7th Dose : (YYYY-MM-DD)
8th Dose : (YYYY-MM-DD)
9th Dose : (YYYY-MM-DD)
10th Dose : (YYYY-MM-DD)
11th Dose : (YYYY-MM-DD)

12th Dose : (YYYY-MM-DD)

Randomization

Lille category	Treatment arms	
Complete responder	Not applicable (continue steroid)	
Partial responder	steroid+placebo vs. steroid+G-CSF	
Null responder	placebo vs. G-CSF	

At admission day (admission lab), if MDF>=32 regardless of biopsy or not, day of starting steroid treatment is defined as day 0.

At admission day, if MDF<32 → Day at MDF>=32 is the baseline (Day 0), / if liver biopsy is performed, day at MDF >=32 is the baseline and day of starting steroid treatment is day 0.

Day 1 (Day of starting steroid treatment or most recent date just before steroid use)

Date of test: (YYYY-MM-DD)

Complete blood count

Item	Result	If abnormal, check CS NCS
WBC($\chi 10^3$/μL)		☐ CS ☐ NCS
Neutrophil (%)		☐ CS ☐ NCS
Lymphocyte (%)		☐ CS ☐ NCS
Monocyte (%)		☐ CS ☐ NCS
Hemoglobin(g/dL)		☐ CS ☐ NCS
Platelet($\chi 10^3$/μL)		☐ CS ☐ NCS

Routine Chemistry

Item	Result	If abnormal, check CS NCS
Inorganic P(mg/dl)		☐ CS ☐ NCS
Glucose(mg/dl)		☐ CS ☐ NCS
BUN(mg/dl)		☐ CS ☐ NCS
Creatinine(mg/dl)		☐ CS ☐ NCS
GFR (mL/min)		☐ CS ☐ NCS
Uric acid(mg/dl)		☐ CS ☐ NCS
Cholesterol(mg/dl)		☐ CS ☐ NCS
Calcium(mg/dl)		☐ CS ☐ NCS
Total Protein(g/dl)		☐ CS ☐ NCS
Albumin(g/dl)		☐ CS ☐ NCS
Total bilirubin(mg/dl)		☐ CS ☐ NCS

Alk.Phosphatase(IU/L)		☐ CS ☐ NCS
AST(SGOT)(IU/L)		☐ CS ☐ NCS
ALT(SGPT)(IU/L)		☐ CS ☐ NCS
GGT (IU/L)		☐ CS ☐ NCS
Na (mmol/L)		☐ CS ☐ NCS
K (mmol/L)		☐ CS ☐ NCS

Coagulation test

item	Result	If abnormal, check CS NCS
PT(Sec)		☐ CS ☐ NCS
PT (%)		☐ CS ☐ NCS
PT (INR)		☐ CS ☐ NCS

Day 3

Date of test: (YYYY-MM-DD)

Complete blood count

Item	Result	If abnormal, check CS NCS
WBC(χ10^3/μL)		☐ CS ☐ NCS
Neutrophil (%)		☐ CS ☐ NCS
Lymphocyte (%)		☐ CS ☐ NCS
Monocyte (%)		☐ CS ☐ NCS
Hemoglobin(g/dL)		☐ CS ☐ NCS
Platelet(χ10^3/μL)		☐ CS ☐ NCS

Routine Chemistry

Item	Result	If abnormal, check CS NCS
Inorganic P(mg/dl)		☐ CS ☐ NCS
Glucose(mg/dl)		☐ CS ☐ NCS
BUN(mg/dl)		☐ CS ☐ NCS
Creatinine(mg/dl)		☐ CS ☐ NCS
GFR (mL/min)		☐ CS ☐ NCS
Uric acid(mg/dl)		☐ CS ☐ NCS
Cholesterol(mg/dl)		☐ CS ☐ NCS
Calcium(mg/dl)		☐ CS ☐ NCS
Total Protein(g/dl)		☐ CS ☐ NCS
Albumin(g/dl)		☐ CS ☐ NCS
Total bilirubin(mg/dl)		☐ CS ☐ NCS

Alk.Phosphatase(IU/L)		☐ CS ☐ NCS
AST(SGOT)(IU/L)		☐ CS ☐ NCS
ALT(SGPT)(IU/L)		☐ CS ☐ NCS
GGT (IU/L)		☐ CS ☐ NCS
Na (mmol/L)		☐ CS ☐ NCS
K (mmol/L)		☐ CS ☐ NCS

Coagulation test

item	Result	If abnormal, check CS NCS
PT(Sec)		☐ CS ☐ NCS
PT (%)		☐ CS ☐ NCS
PT (INR)		☐ CS ☐ NCS

Day 7:

Date of test: (YYYY-MM-DD)

Complete blood count

Item	Result	If abnormal, check CS NCS
WBC(χ10^3/μL)		☐ CS ☐ NCS
Neutrophil (%)		☐ CS ☐ NCS
Lymphocyte (%)		☐ CS ☐ NCS
Monocyte (%)		☐ CS ☐ NCS
Hemoglobin(g/dL)		☐ CS ☐ NCS
Platelet(χ10^3/μL)		☐ CS ☐ NCS

Routine Chemistry

Item	Result	If abnormal, check CS NCS
Inorganic P(mg/dl)		☐ CS ☐ NCS
Glucose(mg/dl)		☐ CS ☐ NCS
BUN(mg/dl)		☐ CS ☐ NCS
Creatinine(mg/dl)		☐ CS ☐ NCS
GFR (mL/min)		☐ CS ☐ NCS
Uric acid(mg/dl)		☐ CS ☐ NCS
Cholesterol(mg/dl)		☐ CS ☐ NCS
Calcium(mg/dl)		☐ CS ☐ NCS
Total Protein(g/dl)		☐ CS ☐ NCS
Albumin(g/dl)		☐ CS ☐ NCS
Total bilirubin(mg/dl)		☐ CS ☐ NCS

Alk.Phosphatase(IU/L)		☐ CS ☐ NCS
AST(SGOT)(IU/L)		☐ CS ☐ NCS
ALT(SGPT)(IU/L)		☐ CS ☐ NCS
GGT (IU/L)		☐ CS ☐ NCS
Na (mmol/L)		☐ CS ☐ NCS
K (mmol/L)		☐ CS ☐ NCS

Coagulation test

item	Result	If abnormal, check CS NCS
PT(Sec)		☐ CS ☐ NCS
PT (%)		☐ CS ☐ NCS
PT (INR)		☐ CS ☐ NCS

Sampling for research

☐ Blood ☐ No

Clinical evaluation

Item,	Result	If abnormal, check CS NCS
Systolic BP (mmHg)		
Diastolic BP (mmHg)		
Pulse rate (bpm)		
Pressor<br>☐Y ☐N	☐Dopamine (dose__ μg/min _)<br>☐Dobutamine (dose__ μg/min _)<br>☐Terlipressin (dose__mg_)	

	☐Epinephrine (dose__ μg/min _)<br>☐Norepinephrine (dose__ μg/min _)	
PaO_2<br>☐Y ☐N	( )mmHg	
SpO_2<br>☐Y ☐N	( ) %	
O_2 supply	☐No ☐Yes ( ) L/min<br>☐ facial mask ☐ nasal prong	
Mechanical ventilation	☐Yes (FiO_2:) ☐No	
Respiratory rate (/min)		
Body temperature (°C)		
Infection at admission	☐Spontaneous bacteremia<br>☐SBP<br>☐Lower respiratory tract infection<br>☐C. difficile<br>☐bacterial enterocolitis<br>☐skin infection<br>☐UTI<br>☐intraabdominal infection<br>☐secondary bacterial peritonitis	
Gastrointestinal hemorrhage (<br>☐Not done ☐Done	☐No ☐Yes(☐Esophageal varix<br>☐Gastric varix<br>☐Portal hypertensive gastropathy<br>☐Gastric ulcer<br>☐Duodenal ulcer	

	□Mallory-Weiss<br>□Angiodysplasia<br>□etc (______)	
Encephalopathy	□No □Yes (grade__ [1-4])	
Dialysis / renal replacement therapy	□Not done □Done	
**Lille score**	□Complete responder(≤0.16)<br>□Partial responder (0.16<Lille<0.56)<br>□Null responder(≥0.56)	

Day 9

Date of test: (YYYY-MM-DD)

Complete blood count

Item	Result	If abnormal, check CS NCS
WBC($\chi 10^3/\mu L$)		☐ CS ☐ NCS
Neutrophil (%)		☐ CS ☐ NCS
Lymphocyte (%)		☐ CS ☐ NCS
Monocyte (%)		☐ CS ☐ NCS
Hemoglobin(g/dL)		☐ CS ☐ NCS
Platelet($\chi 10^3/\mu L$)		☐ CS ☐ NCS

Routine Chemistry

Item	Result	If abnormal, check CS NCS
Inorganic P(mg/dl)		☐ CS ☐ NCS
Glucose(mg/dl)		☐ CS ☐ NCS
BUN(mg/dl)		☐ CS ☐ NCS
Creatinine(mg/dl)		☐ CS ☐ NCS
GFR (mL/min)		☐ CS ☐ NCS
Uric acid(mg/dl)		☐ CS ☐ NCS
Cholesterol(mg/dl)		☐ CS ☐ NCS
Calcium(mg/dl)		☐ CS ☐ NCS
Total Protein(g/dl)		☐ CS ☐ NCS
Albumin(g/dl)		☐ CS ☐ NCS
Total bilirubin(mg/dl)		☐ CS ☐ NCS

Alk.Phosphatase(IU/L)		☐ CS ☐ NCS
AST(SGOT)(IU/L)		☐ CS ☐ NCS
ALT(SGPT)(IU/L)		☐ CS ☐ NCS
GGT (IU/L)		☐ CS ☐ NCS
Na (mmol/L)		☐ CS ☐ NCS
K (mmol/L)		☐ CS ☐ NCS

Coagulation test

item	Result	If abnormal, check CS NCS
PT(Sec)		☐ CS ☐ NCS
PT (%)		☐ CS ☐ NCS
PT (INR)		☐ CS ☐ NCS

Day 11

Date of test: (YYYY-MM-DD)

Complete blood count

Item	Result	If abnormal, check CS NCS
WBC($\chi 10^3$/μL)		☐ CS ☐ NCS
Neutrophil (%)		☐ CS ☐ NCS
Lymphocyte (%)		☐ CS ☐ NCS
Monocyte (%)		☐ CS ☐ NCS
Hemoglobin(g/dL)		☐ CS ☐ NCS
Platelet($\chi 10^3$/μL)		☐ CS ☐ NCS

Routine Chemistry

Item	Result	If abnormal, check CS NCS
Inorganic P(mg/dl)		☐ CS ☐ NCS
Glucose(mg/dl)		☐ CS ☐ NCS
BUN(mg/dl)		☐ CS ☐ NCS
Creatinine(mg/dl)		☐ CS ☐ NCS
GFR (mL/min)		☐ CS ☐ NCS
Uric acid(mg/dl)		☐ CS ☐ NCS
Cholesterol(mg/dl)		☐ CS ☐ NCS
Calcium(mg/dl)		☐ CS ☐ NCS
Total Protein(g/dl)		☐ CS ☐ NCS
Albumin(g/dl)		☐ CS ☐ NCS
Total bilirubin(mg/dl)		☐ CS ☐ NCS
Alk.Phosphatase(IU/L)		☐ CS ☐ NCS

AST(SGOT)(IU/L)		☐ CS ☐ NCS
ALT(SGPT)(IU/L)		☐ CS ☐ NCS
GGT (IU/L)		☐ CS ☐ NCS
Na (mmol/L)		☐ CS ☐ NCS
K (mmol/L)		☐ CS ☐ NCS

Coagulation test

item	Result	If abnormal, check CS NCS
PT(Sec)		☐ CS ☐ NCS
PT (%)		☐ CS ☐ NCS
PT (INR)		☐ CS ☐ NCS

Day 14

Date of test: (YYYY-MM-DD)

Complete blood count

Item	Result	If abnormal, check CS NCS
WBC(χ10^3/μL)		☐ CS ☐ NCS
Neutrophil (%)		☐ CS ☐ NCS
Lymphocyte (%)		☐ CS ☐ NCS
Monocyte (%)		☐ CS ☐ NCS
Hemoglobin(g/dL)		☐ CS ☐ NCS
Platelet(χ10^3/μL)		☐ CS ☐ NCS

Routine Chemistry

Item	Result	If abnormal, check CS NCS
Inorganic P(mg/dl)		☐ CS ☐ NCS
Glucose(mg/dl)		☐ CS ☐ NCS
BUN(mg/dl)		☐ CS ☐ NCS
Creatinine(mg/dl)		☐ CS ☐ NCS
GFR (mL/min)		☐ CS ☐ NCS
Uric acid(mg/dl)		☐ CS ☐ NCS
Cholesterol(mg/dl)		☐ CS ☐ NCS
Calcium(mg/dl)		☐ CS ☐ NCS
Total Protein(g/dl)		☐ CS ☐ NCS
Albumin(g/dl)		☐ CS ☐ NCS
Total bilirubin(mg/dl)		☐ CS ☐ NCS
Alk.Phosphatase(IU/L)		☐ CS ☐ NCS

AST(SGOT)(IU/L)		☐ CS ☐ NCS
ALT(SGPT)(IU/L)		☐ CS ☐ NCS
GGT (IU/L)		☐ CS ☐ NCS
Na (mmol/L)		☐ CS ☐ NCS
K (mmol/L)		☐ CS ☐ NCS

Coagulation test

item	Result	If abnormal, check CS NCS
PT(Sec)		☐ CS ☐ NCS
PT (%)		☐ CS ☐ NCS
PT (INR)		☐ CS ☐ NCS

Day 17

Date of test: (YYYY-MM-DD)

Complete blood count

Item	Result	If abnormal, check CS NCS
WBC($\chi 10^3/\mu L$)		☐ CS ☐ NCS
Neutrophil (%)		☐ CS ☐ NCS
Lymphocyte (%)		☐ CS ☐ NCS
Monocyte (%)		☐ CS ☐ NCS
Hemoglobin(g/dL)		☐ CS ☐ NCS
Platelet($\chi 10^3/\mu L$)		☐ CS ☐ NCS

Routine Chemistry

Item	Result	If abnormal, check CS NCS
Inorganic P(mg/dl)		☐ CS ☐ NCS
Glucose(mg/dl)		☐ CS ☐ NCS
BUN(mg/dl)		☐ CS ☐ NCS
Creatinine(mg/dl)		☐ CS ☐ NCS
GFR (mL/min)		☐ CS ☐ NCS
Uric acid(mg/dl)		☐ CS ☐ NCS
Cholesterol(mg/dl)		☐ CS ☐ NCS
Calcium(mg/dl)		☐ CS ☐ NCS
Total Protein(g/dl)		☐ CS ☐ NCS
Albumin(g/dl)		☐ CS ☐ NCS
Total bilirubin(mg/dl)		☐ CS ☐ NCS
Alk.Phosphatase(IU/L)		☐ CS ☐ NCS

AST(SGOT)(IU/L)		☐ CS ☐ NCS
ALT(SGPT)(IU/L)		☐ CS ☐ NCS
GGT (IU/L)		☐ CS ☐ NCS
Na (mmol/L)		☐ CS ☐ NCS
K (mmol/L)		☐ CS ☐ NCS

Coagulation test

item	Result	If abnormal, check CS NCS
PT(Sec)		☐ CS ☐ NCS
PT (%)		☐ CS ☐ NCS
PT (INR)		☐ CS ☐ NCS

Day 20

Date of test: (YYYY-MM-DD)

Complete blood count

Item	Result	If abnormal, check CS NCS
WBC(χ10^3/μL)		☐ CS ☐ NCS
Neutrophil (%)		☐ CS ☐ NCS
Lymphocyte (%)		☐ CS ☐ NCS
Monocyte (%)		☐ CS ☐ NCS
Hemoglobin(g/dL)		☐ CS ☐ NCS
Platelet(χ10^3/μL)		☐ CS ☐ NCS

Routine Chemistry

Item	Result	If abnormal, check CS NCS
Inorganic P(mg/dl)		☐ CS ☐ NCS
Glucose(mg/dl)		☐ CS ☐ NCS
BUN(mg/dl)		☐ CS ☐ NCS
Creatinine(mg/dl)		☐ CS ☐ NCS
GFR (mL/min)		☐ CS ☐ NCS
Uric acid(mg/dl)		☐ CS ☐ NCS
Cholesterol(mg/dl)		☐ CS ☐ NCS
Calcium(mg/dl)		☐ CS ☐ NCS
Total Protein(g/dl)		☐ CS ☐ NCS
Albumin(g/dl)		☐ CS ☐ NCS
Total bilirubin(mg/dl)		☐ CS ☐ NCS
Alk.Phosphatase(IU/L)		☐ CS ☐ NCS

AST(SGOT)(IU/L)		☐ CS ☐ NCS
ALT(SGPT)(IU/L)		☐ CS ☐ NCS
GGT (IU/L)		☐ CS ☐ NCS
Na (mmol/L)		☐ CS ☐ NCS
K (mmol/L)		☐ CS ☐ NCS

Coagulation test

item	Result	If abnormal, check CS NCS
PT(Sec)		☐ CS ☐ NCS
PT (%)		☐ CS ☐ NCS
PT (INR)		☐ CS ☐ NCS

Day 23

Date of test: (YYYY-MM-DD)

Complete blood count

Item	Result	If abnormal, check CS NCS
WBC($\chi 10^3/\mu L$)		☐ CS ☐ NCS
Neutrophil (%)		☐ CS ☐ NCS
Lymphocyte (%)		☐ CS ☐ NCS
Monocyte (%)		☐ CS ☐ NCS
Hemoglobin(g/dL)		☐ CS ☐ NCS
Platelet($\chi 10^3/\mu L$)		☐ CS ☐ NCS

Routine Chemistry

Item	Result	If abnormal, check CS NCS
Inorganic P(mg/dl)		☐ CS ☐ NCS
Glucose(mg/dl)		☐ CS ☐ NCS
BUN(mg/dl)		☐ CS ☐ NCS
Creatinine(mg/dl)		☐ CS ☐ NCS
GFR (mL/min)		☐ CS ☐ NCS
Uric acid(mg/dl)		☐ CS ☐ NCS
Cholesterol(mg/dl)		☐ CS ☐ NCS
Calcium(mg/dl)		☐ CS ☐ NCS
Total Protein(g/dl)		☐ CS ☐ NCS
Albumin(g/dl)		☐ CS ☐ NCS
Total bilirubin(mg/dl)		☐ CS ☐ NCS
Alk.Phosphatase(IU/L)		☐ CS ☐ NCS

AST(SGOT)(IU/L)		☐ CS ☐ NCS
ALT(SGPT)(IU/L)		☐ CS ☐ NCS
GGT (IU/L)		☐ CS ☐ NCS
Na (mmol/L)		☐ CS ☐ NCS
K (mmol/L)		☐ CS ☐ NCS

Coagulation test

item	Result	If abnormal, check CS NCS
PT(Sec)		☐ CS ☐ NCS
PT (%)		☐ CS ☐ NCS
PT (INR)		☐ CS ☐ NCS

Day 26

Date of test: (YYYY-MM-DD)

Complete blood count

Item	Result	If abnormal, check CS NCS
WBC($\chi10^3/\mu L$)		☐ CS ☐ NCS
Neutrophil (%)		☐ CS ☐ NCS
Lymphocyte (%)		☐ CS ☐ NCS
Monocyte (%)		☐ CS ☐ NCS
Hemoglobin(g/dL)		☐ CS ☐ NCS
Platelet($\chi10^3/\mu L$)		☐ CS ☐ NCS

Routine Chemistry

Item	Result	If abnormal, check CS NCS
Inorganic P(mg/dl)		☐ CS ☐ NCS
Glucose(mg/dl)		☐ CS ☐ NCS
BUN(mg/dl)		☐ CS ☐ NCS
Creatinine(mg/dl)		☐ CS ☐ NCS
GFR (mL/min)		☐ CS ☐ NCS
Uric acid(mg/dl)		☐ CS ☐ NCS
Cholesterol(mg/dl)		☐ CS ☐ NCS
Calcium(mg/dl)		☐ CS ☐ NCS
Total Protein(g/dl)		☐ CS ☐ NCS
Albumin(g/dl)		☐ CS ☐ NCS
Total bilirubin(mg/dl)		☐ CS ☐ NCS
Alk.Phosphatase(IU/L)		☐ CS ☐ NCS

AST(SGOT)(IU/L)		☐ CS ☐ NCS
ALT(SGPT)(IU/L)		☐ CS ☐ NCS
GGT (IU/L)		☐ CS ☐ NCS
Na (mmol/L)		☐ CS ☐ NCS
K (mmol/L)		☐ CS ☐ NCS

Coagulation test

item	Result	If abnormal, check CS NCS
PT(Sec)		☐ CS ☐ NCS
PT (%)		☐ CS ☐ NCS
PT (INR)		☐ CS ☐ NCS

Day 29

Date of test: (YYYY-MM-DD)

Complete blood count

Item	Result	If abnormal, check CS NCS
WBC(χ10^3/μL)		☐ CS ☐ NCS
Neutrophil (%)		☐ CS ☐ NCS
Lymphocyte (%)		☐ CS ☐ NCS
Monocyte (%)		☐ CS ☐ NCS
Hemoglobin(g/dL)		☐ CS ☐ NCS
Platelet(χ10^3/μL)		☐ CS ☐ NCS

Routine Chemistry

Item	Result	If abnormal, check CS NCS
Inorganic P(mg/dl)		☐ CS ☐ NCS
Glucose(mg/dl)		☐ CS ☐ NCS
BUN(mg/dl)		☐ CS ☐ NCS
Creatinine(mg/dl)		☐ CS ☐ NCS
GFR (mL/min)		☐ CS ☐ NCS
Uric acid(mg/dl)		☐ CS ☐ NCS
Cholesterol(mg/dl)		☐ CS ☐ NCS
Calcium(mg/dl)		☐ CS ☐ NCS
Total Protein(g/dl)		☐ CS ☐ NCS
Albumin(g/dl)		☐ CS ☐ NCS
Total bilirubin(mg/dl)		☐ CS ☐ NCS
Alk.Phosphatase(IU/L)		☐ CS ☐ NCS

AST(SGOT)(IU/L)		☐ CS ☐ NCS
ALT(SGPT)(IU/L)		☐ CS ☐ NCS
GGT (IU/L)		☐ CS ☐ NCS
Na (mmol/L)		☐ CS ☐ NCS
K (mmol/L)		☐ CS ☐ NCS

Coagulation test

item	Result	If abnormal, check CS NCS
PT(Sec)		☐ CS ☐ NCS
PT (%)		☐ CS ☐ NCS
PT (INR)		☐ CS ☐ NCS

Day 32

Date of test: (YYYY-MM-DD)

Complete blood count

Item	Result	If abnormal, check CS NCS
WBC($\chi10^3/\mu L$)		☐ CS ☐ NCS
Neutrophil (%)		☐ CS ☐ NCS
Lymphocyte (%)		☐ CS ☐ NCS
Monocyte (%)		☐ CS ☐ NCS
Hemoglobin(g/dL)		☐ CS ☐ NCS
Platelet($\chi10^3/\mu L$)		☐ CS ☐ NCS

Routine Chemistry

Item	Result	If abnormal, check CS NCS
Inorganic P(mg/dl)		☐ CS ☐ NCS
Glucose(mg/dl)		☐ CS ☐ NCS
BUN(mg/dl)		☐ CS ☐ NCS
Creatinine(mg/dl)		☐ CS ☐ NCS
GFR (mL/min)		☐ CS ☐ NCS
Uric acid(mg/dl)		☐ CS ☐ NCS
Cholesterol(mg/dl)		☐ CS ☐ NCS
Calcium(mg/dl)		☐ CS ☐ NCS
Total Protein(g/dl)		☐ CS ☐ NCS
Albumin(g/dl)		☐ CS ☐ NCS
Total bilirubin(mg/dl)		☐ CS ☐ NCS
Alk.Phosphatase(IU/L)		☐ CS ☐ NCS

AST(SGOT)(IU/L)		☐ CS ☐ NCS
ALT(SGPT)(IU/L)		☐ CS ☐ NCS
GGT (IU/L)		☐ CS ☐ NCS
Na (mmol/L)		☐ CS ☐ NCS
K (mmol/L)		☐ CS ☐ NCS

Coagulation test

item	Result	If abnormal, check CS NCS
PT(Sec)		☐ CS ☐ NCS
PT (%)		☐ CS ☐ NCS
PT (INR)		☐ CS ☐ NCS

Day 35

Date of test: (YYYY-MM-DD)

Complete blood count

Item	Result	If abnormal, check CS NCS
WBC(χ10^3/μL)		☐ CS ☐ NCS
Neutrophil (%)		☐ CS ☐ NCS
Lymphocyte (%)		☐ CS ☐ NCS
Monocyte (%)		☐ CS ☐ NCS
Hemoglobin(g/dL)		☐ CS ☐ NCS
Platelet(χ10^3/μL)		☐ CS ☐ NCS

Routine Chemistry

Item	Result	If abnormal, check CS NCS
Inorganic P(mg/dl)		☐ CS ☐ NCS
Glucose(mg/dl)		☐ CS ☐ NCS
BUN(mg/dl)		☐ CS ☐ NCS
Creatinine(mg/dl)		☐ CS ☐ NCS
GFR (mL/min)		☐ CS ☐ NCS
Uric acid(mg/dl)		☐ CS ☐ NCS
Cholesterol(mg/dl)		☐ CS ☐ NCS
Calcium(mg/dl)		☐ CS ☐ NCS
Total Protein(g/dl)		☐ CS ☐ NCS
Albumin(g/dl)		☐ CS ☐ NCS
Total bilirubin(mg/dl)		☐ CS ☐ NCS

Alk.Phosphatase(IU/L)		☐ CS ☐ NCS
AST(SGOT)(IU/L)		☐ CS ☐ NCS
ALT(SGPT)(IU/L)		☐ CS ☐ NCS
GGT (IU/L)		☐ CS ☐ NCS
Na (mmol/L)		☐ CS ☐ NCS
K (mmol/L)		☐ CS ☐ NCS

Coagulation test

item	Result	If abnormal, check CS NCS
PT(Sec)		☐ CS ☐ NCS
PT (%)		☐ CS ☐ NCS
PT (INR)		☐ CS ☐ NCS

Urinalysis

Specimen	Item	Result	If abnormal, check CS NCS
Urinalysis	WBC(/HPF)		☐ CS ☐ NCS
	RBC(/HPF)		☐ CS ☐ NCS
	cast		☐ CS ☐ NCS
	bacteria		☐ CS ☐ NCS
	bilirubin		☐ CS ☐ NCS
	nitrite		☐ CS ☐ NCS
	Na/K/Cl(mmol/L)		☐ CS ☐ NCS

Sampling for research

□ Blood □ Liver tissue □ No

Clinical evaluation

Item	Result	If abnormal, check CS NCS
Systolic BP (mmHg)		
Diastolic BP (mmHg)		
Pulse rate (bpm)		
Pressor	□Dopamine (dose__ µg/min _)<br>□Dobutamine (dose_µg/min)<br>□Terlipressin (dose__mg_)<br>□Epinephrine (dose___µg/min)<br>□Norepinephrine (dose__µg/min _)	
PaO_2<br>□Y □N	( )mmHg	
SpO_2<br>□Y □N	( ) %	
O_2 supply	( ) L/min<br>□ facial mask □ nasal prong	
Mechanical ventilation	□Yes (FiO_2:) □No	
Respiratory rate (/min)		
Body temperature (°C)		
Infection at admission	□Spontaneous bacteremia □ SBP □Lower respiratory tract infection □C. difficile □	

	bacterial enterocolitis ☐skin infection ☐UTI ☐ intraabdominal infection ☐secondary bacterial peritonitis	
Gastrointestinal hemorrhage ☐Not done ☐Done	☐No ☐Yes (☐Esophageal varix ☐Gastric varix ☐Portal hypertensive gastropathy ☐Gastric ulcer ☐ Duodenal ulcer ☐Mallory-Weiss ☐Angiodysplasia ☐etc (______)	
Encephalopathy	☐No ☐Yes (grade__ [1-4])	
Dialysis / renal replacement therapy	☐Not done ☐Done	

Imaging test: ☐ Yes ☐ No

Date: (YYYY-MM-DD)

Result:

Tissue (histologic evaluation, HVPG)

Item	Result	If abnormal, check CS NCS
HVPG<br>☐Y ☐N<br>(YYYY-MM-DD)	( )mmHg	
Histologic scoring	hepatocellular damage/ballooning (0=mild;	

(transjugular or percutaneous liver biopsy)<br>☐Y ☐N<br>(YYYY-MM-DD)	1=marked)	
	Mallory bodies (0=absent; 1=present)	
	Polymorphonuclear (PMN) infiltration (0=mild; 1=moderate/severe)	
	Steatosis (0, <33%; 1, 33 - 66%; 2, >66%)	
	Lobular fibrosis (0=no fibrosis or zone 3; 1=fibrosis in zone 2+3; 2=panlobular fibrosis),	
	Fibrosis stage (0=no fibrosis or portal fibrosis; 1= expansive periportal fibrosis; 2= bridging fibrosis and cirrhosis)	
	Megamitochondria (0=no; 1=yes)	
	Bilirubinostasis (0=none; 1=hepatocellular bilirubinostasis; 2=canalicular or ductular bilirubinostasis; 3=hepatocellular + canalicular or ductular bilirubinostasis)	

< Re-evaluation items during hospitalization >

1. Nosocomial or second infection (Recorded each time it occurs)

- date: YYYY-MM-DD
- Admission 48 hours - Infection-related symptoms until discharge

☐cough ☐sputum production ☐dyspnea ☐pleuritic pain ☐diarrhea

- Infection-related physical examination and examination findings

☐rale ☐crepitation ☐shivering ☐cellulitis ☐intraabdominal infection evidence (☐diverticulitis ☐appendicitis ☐cholangitis) ☐C.difficile antibody

- Type of infection

☐Spontaneous bacteremia ☐SBP ☐Lower respiratory tract infection ☐C. difficile ☐bacterial enterocolitis ☐skin infection ☐UTI ☐intraabdominal infection ☐secondary bacterial peritonitis

-Presence of SIRS: Body temperature ( )°C Heart rate ( ) /min RR ( )/min WBC(date of infection determination) ( )/μL

2. Presence of ACLF (acute-on-chronic liver failure)

1) Liver (bilirubin)
- Date of aggravation: YYYY-MM-DD
- value: ( )mg/dL

2) Kidney (creatinine)
- Date of aggravation: YYYY-MM-DD
- value: ( )mg/dL
- new Renal Replacement Therapy: ☐None ☐Done

3) Cerebral (HE grade)
- Date of aggravation: YYYY-MM-DD
- Grade (I-IV)

4) Coagulation

- Date of aggravation: YYYY-MM-DD
- value: INR (   )   platelet [if < 20,000/μL] (   )/μL

5) Circulation (BP)
- Date of aggravation: YYYY-MM-DD
- value: sBP (   )   dBP (   )mmHg
- new pressor: □Dopamine (dose___ μg/min) □Dobutamine (dose___ μg/min) □Terlipressin (dose__mg_)

□Epinephrine (dose___ μg/min) □Norepinephrine (dose___ μg/min)

6) Lung
- Date of aggravation: YYYY-MM-DD
- PaO_2 (ABGA)   (   )mmHg SpO_2 (   )%
- O_2 (   )L/min □ facial mask □ nasal prong
- Mechanical ventilation: □Yes (FiO_2:   ) □No

3. Endpoint

1) During hospitalization
- Date: YYYY-MM-DD
- Endpoint: □ Survival(recovery) □ Survival (transfer) □ Death □ Transplantation (LDLT__(donor:__)/DDLT__)

2) After discharge
- Last visit date (or date of death): YYYY-MM-DD
- Endpoint: □ Survival □ Death □ Unknown

3) Cause of death

□Liver failure □ACLF □Infection/sepsis □Gastrointestinal bleeding □Hepatorenal syndrome □Etc(describe)____

Adverse Event ☐Yes ☐No Subject number: Initial:

Adverse Event	Date of occurence (YYYY/MM/DD)	Date of lost (YYYY/MM/DD)	Grade A))	Serious (B)	Result (C)	Causal relationship(D)	Treatment(E)	IP interruption (F)	IP restart date (YYYY/MM/DD)
	/ /	/ / (❑continuous)							/ /
	/ /	/ / (❑continuous)							/ /
	/ /	/ / (❑continuous)							/ /
	/ /	/ / (❑continuous)							/ /
	/ /	/ / (❑continuous)							/ /
	/ /	/ / (❑continuous)							/ /
	/ /	/ / (❑continuous)							/ /

**Grade (A)**	**1=Mild, 2=Moderate, 3=Severe**
**Serious (B)**	**1=not serious, 2=Serious,** ***(if SAE, please report)***
**Result (C)**	**1=recovery, without complication, 2= recovery, with complication, 3=continous, no need for follow-up, 4= continous, follow-up loss, 5=death**
**Causal relationship (D)**	**1=relation (+), 2= relation (-), 3=Impossible evaluation, 4=NA**
**Treatment (E)**	**1=No treatment, 2=Medication, 3=Non-medical treatment, 4=Admission/prologation of admission**
**IP interruption**	**1= Permanent interruption, 2= Temporary interruption, 3=No interruption, 4=NA**

(F)

**Drug (Ingredients)**	**Purpose**	**Daily dose**	**Dosage unit**[1]	**Route of administration**[2]	**Start date of administration (DD/MMM/YYYY)**	**End date of administration (DD/MMM/YYYY)**	**During administration**
							☐
							☐
							☐
							☐
							☐
							☐
							☐
							☐

**unit Reference number**	**01**	mg	**02**	g	**03**	mcg	**04**	mL	**05**	L
	**06**	T(Tablet)	**07**	C(Capsule)	**88**	NA(not applicable)	**97**	etc	**99**	UK

**Route Reference number**	**01**	oral: PO	**02**	sublingual: SL	**03**	Intramuscular :IM	**04**	intravenous: IV
	**05**	subcutaneous: SC	**06**	Inhalation	**07**	Skin application	**08**	Mucosal application
	**09**	Rectal administration	**10**	etc	**88**	NA	**99**	UK

Concomitant medication ☐Yes ☐ No Subject number: Initial:

Unscheduled visit

Date of visit:

Subject number: Initial:

1. Reason for visit
   - ☐ Adverse event
   - ☐ If medical treatment is required after clinical examination
   - ☐ etc (reason :

2. Bwt: ☐Yes ☐ No , ____________kg
3. Vital sign ☐Yes ☐ No

BT: ℃ PR: /min RR: /min S.B.P/D.B.P: mmHg

4. ECOG Performance Status ☐ 0 ☐ 1 ☐ 2 ☐ 3 ☐ 4
5. Identified concomitant medication:
6. Identified adverse reactions:
7. Performed lab tests:

Serious adverse event

Subject number: Initial: Age: Sex : organization :

Study Title: **Efficacy of granulocyte colony stimulating factor in patients with severe alcoholic hepatitis with partial or null response to steroid: A randomized, double-blind, placebo-controlled, nationwide multi-center study (GRACIAH study)**

Version Number: 1.1

Research Director: Affilliation:

Investigator-Initiated Trial (IIT) manager: Tel:

Date of report: YYYY/MM/DD

Report classification: ☐ initial report ☐ Follow-up report ☐ Final report

Follow-up report, #____________

Adverse event:

Date: YYYY/MM/DD End of adverse event: YYYY/MM/DD

- ☐ Death
- ☐ Results in persistent or significant disability or incapacity involving a congenital anomaly or birth defect
- ☐ Life-threatening events
- ☐ Any other important medical event that carries a real, not a hypothetical risk of one of the outcomes above

Past medical history:

Concomitant medication:

Lab tests:

Details of Adverse events (Describe in detail of adverse events, clinical courses)

Action for Adverse Reactions (Describe in detail of treatment, results, and follow-up management)

★ Whether or not information about serious adverse events has been forwarded to the sponsor

☐Yes ☐No ☐N/A

★ Whether a research plan change is necessary?

☐Yes ☐No

★ Whether any change in consent form is required?

☐Yes (Attached form of revised written consent form) ☐No

Date: YYYY/MM/DD

Doctor:

Writer:

Comment:

Follow-up visit (Day 60, Day 90, Day 120, Day 150, and Day 180)

* If the hospitalization is continued until the above point, repeat the following items every 1 week until the time of discharge, and follow the above point after discharge

Clinical assessment

1. Alcohol intake: follow-up AUDIT-K ☐Yes ☐No

Date of Laboratory test:

Complete blood count

Item	Result	If abnormal results
WBC($\chi 10^3/\mu L$)		☐ CS ☐ NCS
Neutrophil (%)		☐ CS ☐ NCS
Lymphocyte (%)		☐ CS ☐ NCS
Monocyte (%)		☐ CS ☐ NCS
Hemoglobin(g/dL)		☐ CS ☐ NCS
Platelet($\chi 10^3/\mu L$)		☐ CS ☐ NCS

Routine chemistry

Item	Result	If abnormal results
Inorganic P(mg/dl)		☐ CS ☐ NCS
Glucose(mg/dl)		☐ CS ☐ NCS
BUN(mg/dl)		☐ CS ☐ NCS
Creatinine(mg/dl)		☐ CS ☐ NCS
GFR (mL/min)		☐ CS ☐ NCS

Uric acid(mg/dl)		☐ CS ☐ NCS
Cholesterol(mg/dl)		☐ CS ☐ NCS
Calcium(mg/dl)		☐ CS ☐ NCS
Total Protein(g/dl)		☐ CS ☐ NCS
Albumin(g/dl)		☐ CS ☐ NCS
Total bilirubin(mg/dl)		☐ CS ☐ NCS
Alk.Phosphatase(IU/L)		☐ CS ☐ NCS
AST(SGOT)(IU/L)		☐ CS ☐ NCS
ALT(SGPT)(IU/L)		☐ CS ☐ NCS
GGT (IU/L)		☐ CS ☐ NCS
Na (mmol/L)		☐ CS ☐ NCS
K (mmol/L)		☐ CS ☐ NCS

Coagulation test

Item	Result	If abnormal results
PT(Sec)		☐ CS ☐ NCS
PT (%)		☐ CS ☐ NCS
PT (INR)		☐ CS ☐ NCS
